# Usefulness of Somatosensory Evoked Potentials in Non-traumatic Spinal Cord Injury in the Absence of MRI Abnormalities: A Case Report

**DOI:** 10.7759/cureus.90526

**Published:** 2025-08-19

**Authors:** Yuki Inami, Masataka Kitabatake, Katsunori Fujii

**Affiliations:** 1 Department of Pediatrics, International University of Health and Welfare Narita Hospital, Narita, JPN; 2 Department of Pediatrics, Asahi General Hospital, Asahi, JPN

**Keywords:** myelopathy, non-traumatic spinal cord injury, sci, seps, somatosensory evoked potentials

## Abstract

Non-traumatic spinal cord injury (ntSCI) in childhood rarely occurs during activities of daily living, such as stretching, surfing, or physical education classes. Its pathogenesis remains to be elucidated; however, excessive spinal extension has been suggested as the causative etiology. A previously healthy 14-year-old Japanese girl ran many shuttles in her physical education class and suddenly experienced back pain, followed by severe muscle weakness in the lower extremities. As she was unable to walk independently, she was admitted to the hospital for investigation. Upon admission, she exhibited left-dominant paralysis of the lower extremities, with an increased left patellar tendon reflex, although the deep sensory and cranial nerves were preserved. Spinal MRI, including diffusion-weighted imaging, revealed no abnormalities on days 1 and 24. However, somatosensory evoked potentials (SEPs) on day 22 showed significant left-sided prolongation of the central conduction time (CCT) between N20 and P38, which was clinically consistent with the side of her left-dominant paralysis. We diagnosed the patient with non-traumatic acute spinal cord injury associated with excessive exercise and initiated rehabilitation immediately. She was gradually able to walk without support, but the abnormality of the SEPs - as the left side prolonged the CCT between N20 and P38 - remained on day 116. We consider that SEPs could be an alternative method for detecting occult spinal lesions in children, especially in cases where spinal MRI fails to demonstrate the lesion responsible for ntSCI.

## Introduction

Non-traumatic spinal cord injury (ntSCI) is a rare myelopathy that occurs during daily activities. This concept includes similar entities such as spontaneous spinal cord infarction [1,2], transient spinal ischemia, ischemic myelopathy, Surfer’s myelopathy [3,4], and spinal cord injury without radiological findings (also called SCIWORA) [5]. The common etiology is thought to be a transient ischemic attack or excessive truncal extension of the spinal cord [1-4]. The clinical manifestations are relatively stereotypical, typically involving back pain followed by muscle weakness in the lower extremities, with or without urinary distention [1-5]. While ntSCI mainly occurs in adults, recent reports have described its occurrence in children during daily exercise activities [6,7].

The epidemiology of ntSCI has been reported worldwide. The incidence rate is 26.9 per million per year in Ireland and 39.8 per million per year in South Korea. A key clinical characteristic is the presence of symptoms originating from the spinal cord without loss of consciousness.

The diagnosis of ntSCI is usually based on clinical manifestations and MRI findings. However, 25% of patients with spontaneous spinal cord infarction show no MRI abnormalities at disease onset [1,2]. Therefore, repeat MRI is recommended for the detection of spinal lesions [1]. In cases where repeat MRI still does not reveal abnormalities, the diagnosis of ntSCI remains difficult.

Treatment is directed at addressing the underlying disease, but if the condition is caused by a blood flow disorder, there is no fundamental treatment, and rehabilitation becomes the main focus.

Somatosensory evoked potentials (SEPs) physiologically reflect electrical activity from peripheral sensory nerves through the posterior spinal column to the primary sensory cortex after tactile stimulation, and they are useful adjunctive methods for detecting occult spinal lesions in children.

Herein, we describe the usefulness of SEPs in a 14-year-old Japanese girl who exhibited an ntSCI without MRI abnormalities. Because diagnostic tools for ntSCI are limited, SEPs could become an adjunctive tool for detecting occult spinal lesions in children. We obtained informed consent to publish this paper from the patient and her parents.

## Case presentation

A previously healthy 14-year-old girl was born at term by normal delivery. She had normal development and no prior clinical history. Family history revealed no siblings, and apart from a father with gastric ulcers and a mother with migraines, there was no evidence of cerebrovascular disease, hypertension, or heart disease. On the day of symptom onset, she attended school in good spirits and actively participated in physical education classes, with no warning signs noted before the onset of symptoms. While running shuttles during a physical education class, she suddenly felt back pain while turning, followed by left-dominant muscle weakness of the lower extremities. She was unable to walk due to left-sided paralysis in the lower extremities and was admitted to our hospital.

On admission, she was alert, but manual muscle testing showed grade 3 strength (fair: the muscle can move the joint through its full range of motion against gravity, but not against any added resistance) in the left quadriceps, hamstrings, and tibialis anterior muscles, and grade 4 strength on the right. The upper extremities were unremarkable. The left patellar tendon reflex was prominently increased, although the Babinski response was negative bilaterally. The sphincter response, deep sensory function, cranial nerves, and cerebellar function were preserved. Because the patient's upper limb motor function was normal but lower limb motor impairment was present, we believe the lesion occurred between C7 and L3.

**Figure 1:**
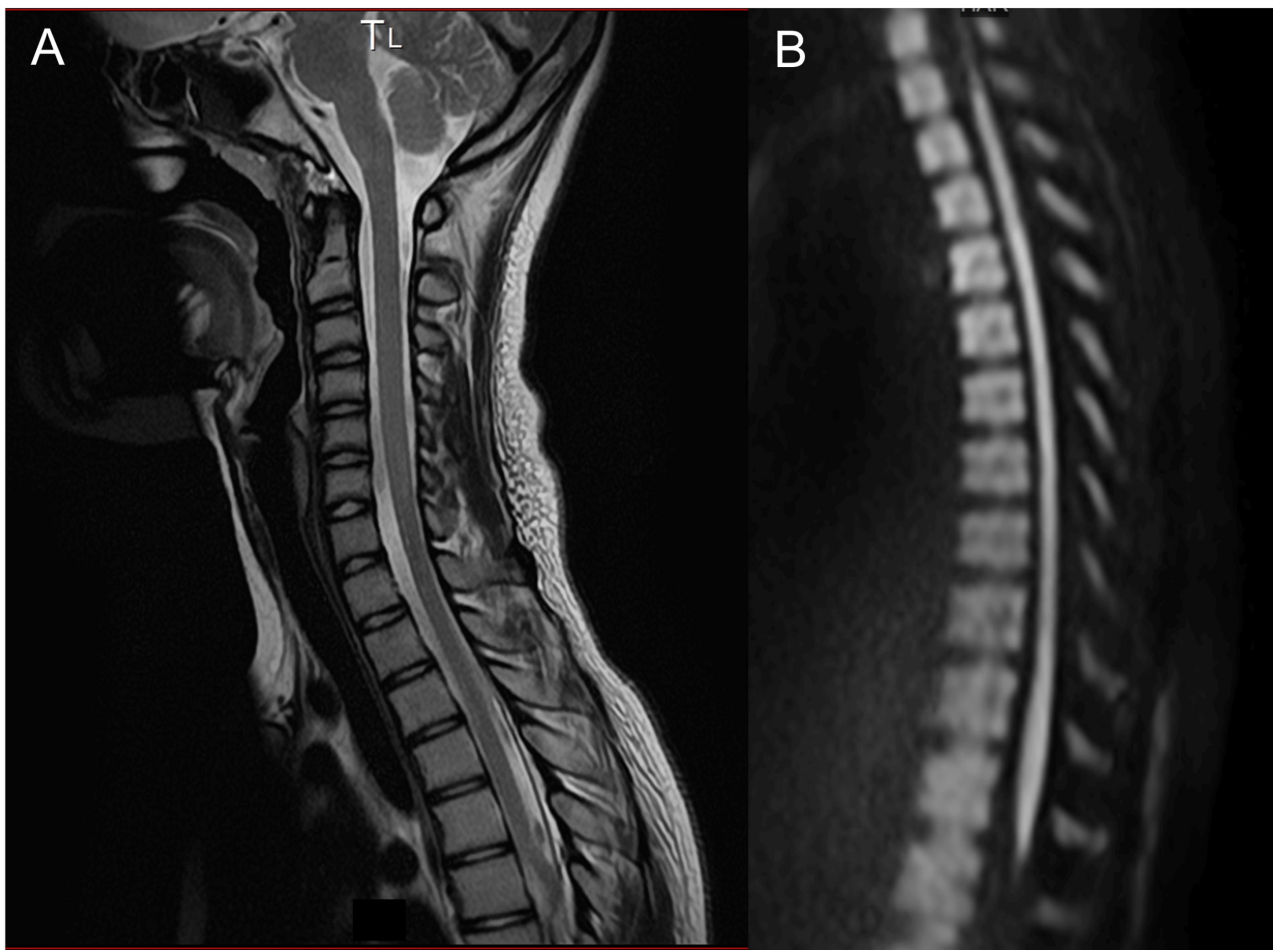
Spinal MRI, including diffusion-weighted imaging, showing no abnormalities on day 1 (A) Sagittal T2-weighted image (cervical-thoracic); (B) sagittal diffusion-weighted image (thoracic-lumbar). No parenchymal abnormalities in the spinal cord were observed.

Spinal MRI, including diffusion-weighted and diffusion tensor imaging, showed no abnormalities on day 1 (Figure 1) and day 24 (data not shown). Radiological findings of the spine, brain MRI, and blood examinations - including metabolic screening - were unremarkable (Table 1).

**Table 1:** Blood tests Blood tests revealed no abnormalities that could explain her symptoms. PT: prothrombin time; aPTT: activated partial thromboplastin time

Variables	On admission	Reference range	Unit
White blood cells	6110	3500-9800	/µL
Hemoglobin	14.8	13.4-17.7	g/dL
Platelet	242	30-362	x 10^3^/µL
PT	108	70-140	%
aPTT	28.5	24-37	sec
Fibrinogen	191	155-300	mg/dL
D-dimer	<0.5	<1.0	µg/mL
Total protein	7.3	6.5-8.0	g/dL
Albumin	4.9	4.0-5.0	g/dL
Aspartate aminotransferase	25	11.0-25.0	IU/L
Alanine aminotransferase	10	6.0-30.0	IU/L
Lactate dehydrogenase	214	124-222	IU/L
Total bilirubin	0.6	0.3-1.4	mg/dL
Creatine kinase	116	35-160	IU/L
Glucose	96	80-120	mg/dL
C-reative protein	<0.01	0-0.3	mg/dL

**Figure 2:**
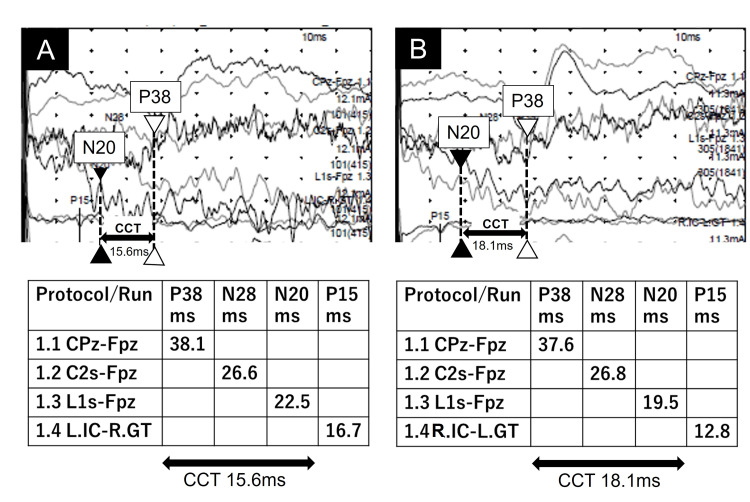
SEPs of this patient on day 22 Somatosensory evoked potentials (SEPs) components and central conduction time (CCT) measurements of the patient (height: 1.67 m). (A) Right SEPs obtained by stimulating the right posterior tibial nerve. (B) Left SEPs obtained by stimulating the left posterior tibial nerve. In the 4-channel montage, the active electrodes are CPz, C2s, L1s, and GT, respectively. Peripheral P15 is referenced to the iliac crest (IC). Cortical P38, brainstem N28, and spinal cord N20 are referenced to Fpz. The solid arrowhead and the vertical dotted line indicate spinal entry time, coinciding with the onset of the spinal N20 component. The open arrowhead and the dotted lines indicate the onset of the cortical P38 component. The CCT onset was measured from the spinal entry time (N20) to the peak of the cortical P38 component. Note that the CCT between the P38 and N20 peaks was prolonged after left-sided stimulation. C2s: spinous process of the 2nd cervical vertebra; L1s: spinous process of the 1st lumbar vertebra; IC: iliac crest; GT: greater trochanter

We diagnosed an ntSCI associated with excessive exercise in running shuttles and started rehabilitation immediately. The differential diagnosis, other than ntSCI, includes periodic paralysis, Guillain-Barré syndrome, chronic idiopathic demyelinating polyradiculopathy, and psychological disorder. We excluded Guillain-Barré syndrome and chronic idiopathic demyelinating polyradiculopathy by electrophysiological examination and clinical symptoms such as increased tendon reflexes. Periodic paralysis was also excluded due to its different onset pattern. Finally, psychological disorders were ruled out based on the consistency of symptoms and physiological test results. We also examined the SEPs on day 22. The posterior tibial nerve was stimulated, revealing that the time from spinal peak N20 to cortical peak P38 [8] was 15.6 ms on the right and 18.1 ms on the left, indicating prolonged central conduction time (CCT) on the left (Figure 2).

**Figure 3:**
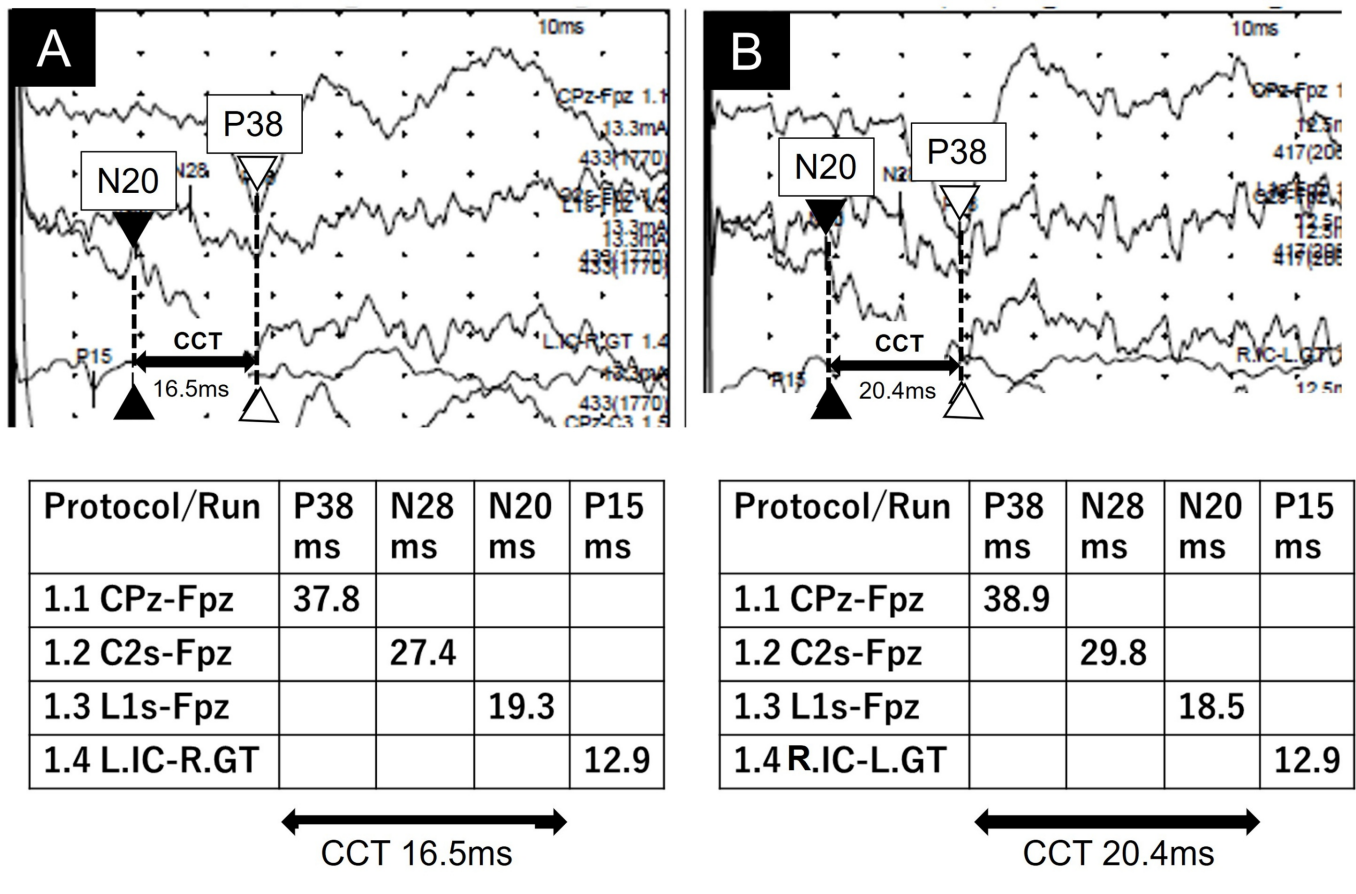
SEPs of this patient on day 116 Somatosensory evoked potentials (SEPs) components and central conduction time (CCT) measurements of the patient (height: 1.67 m). (A) Right SEPs obtained by stimulating the right posterior tibial nerve. (B) Left SEPs obtained by stimulating the left posterior tibial nerve. The active electrodes and references are the same as those used on day 22 (Figure 2). C2s: spinous process of the 2nd cervical vertebra; L1s: spinous process of the 1st lumbar vertebra; IC: iliac crest; GT: greater trochanter

We repeated the SEPs on day 116, during which the time from spinal peak N20 to cortical peak P38 was 15.6 ms on the right and 18.1 ms on the left, confirming the prolonged CCT on the left again (Figure 3).

After rehabilitation, the patient gradually became able to walk without support on day 20 and was discharged on day 25.

## Discussion

In this report, we describe the case of a 14-year-old girl with an acute ntSCI caused by excessive physical activity involving running shuttles. Although repeated spinal MRI did not detect abnormalities, the SEPs demonstrated left-sided prolonged CCT on days 22 and 116. Since these findings coincided with the side of the patient’s left-dominant paralysis, we concluded that SEPs could be a useful adjunctive method to detect occult lesions in patients with ntSCI without MRI abnormalities. SEPs physiologically reflect electrical activity from peripheral sensory nerves through the posterior spinal column to the primary sensory cortex after tactile stimulation [9]. Because SEPs have a small preliminary nature and imperfect interrater reliability, the results should be interpreted with caution, especially in young children [10]. In this patient, we performed SEPs twice - on days 22 and 116 - which showed identical results of left-sided prolongation of CCT between N20 and P38. This prolongation was clinically consistent with left-dominant paralysis.

The normal value of CCT in children has already been reported [8,11]. However, there is a wide range of normal CCT in children due to factors such as height, nerve diameter, and neural myelination [8,11]. Thus, even if the CCT value is within the normal range, a persistent difference between the right and left CCT would be significant in light of the neurological findings observed in the patient.

The reason why the CCT values did not change, even though the patient was able to walk, remains to be elucidated. While SEPs reflect the function of the dorsal columns of the spinal cord, the anterior nuclei that control movement are located in the anterior columns. The exact reason for this is still speculative, but it may reflect differences in the distribution of spinal cord function. In a previous report, SEP results did not necessarily correlate with clinical symptoms [12]. Therefore, we do not consider this to be a contradictory result. Motor evoked potentials obtained by transcranial magnetic stimulation (TMS) are a good indicator of motor function. However, because TMS involves applying magnetic stimulation to the subject's head, the patient refused to undergo the test. Therefore, TMS was not performed in this study.

Spinal MRI occasionally shows no abnormalities at the beginning of ntSCI. In such cases, alternative methods are required to reveal the lesion affecting the spinal cord. Spinal MRI is usually recommended to be performed 48-72 hours after the onset of symptoms. However, in this case, due to various circumstances, it was not possible to perform an MRI within this time frame. Thus, careful consideration is needed regarding the consistency of the MRI with the SEP test. Given the MRI protocol limitations and the single-case design, SEPs may be a useful adjunctive tool when MRI is inconclusive or unavailable, rather than a definitive diagnostic method.

In a previous report, a 30-year-old man with multiple sclerosis exhibited decreased vibratory sensation on the left side, which was not explained by spinal MRI. However, he had abnormal SEPs in the left side and prolonged CCT between N-19 and P37, which was consistent with his decreased vibratory sensation on the left [13]. Given that the spinal MRI of this patient did not show any abnormalities in the dorsal portion of the spinal cord, SEPs were possibly able to detect occult spinal lesions in adult patients. Therefore, SEPs could be an alternative method for detecting spinal lesions without MRI abnormalities.

SEPs have already been reported in patients with traumatic SCI [14]. In those patients, SEPs were used to confirm the damaged lesions or to predict the clinical outcome by scoring the latency and amplitude of SEPs [15]. In our patient, we repeatedly showed the usefulness of SEPs in ntSCI with the absence of MRI abnormalities. This will become an alternative strategy when we cannot detect spinal lesions on MRI and need to determine a therapeutic approach promptly.

SEPs have also been applied to determine prognosis in multiple sclerosis, myoclonus, intraoperative monitoring, and hypoxia-ischemic encephalopathy [16]. In cardiac arrest survivors, SEPs are thought to be accurate predictors of poor outcomes, with high specificity and a low risk of false positives [16]. In addition, bilaterally absent N20 waves from median nerve stimulation in adults and children were suggested to predict poor neurological outcomes, with 88% specificity in cohort studies [17,18]. Because one patient in this study showed that the lack of N20 resulted in a good outcome, the prognostic value of SEPs remains controversial. In addition, the inter-rater reliability in children seems so unstable that careful interpretation of SEPs is necessary [10]. It should also be noted that the N20 elicited by median nerve stimulation reflects the brain cortex, whereas the N20 elicited by posterior tibial nerve stimulation reflects the first lumbar spinal cord [11,19].

The characteristics of childhood SEPs have also been investigated. The latency and amplitude of the evoked potential can be affected by growth changes in children [20]. However, the laterality of CCT is not influenced [8,13]. Thus, our reproducible data - left prolongation of CCT - were valuable because they were consistent with the clinical features.

This case report has some limitations. Since SEPs were first performed on day 22 and the second MRI was on day 24, these assessments were not performed concurrently. This timing mismatch may limit the ability to conclude that SEPs revealed abnormalities missed by MRI.

## Conclusions

We described the case of a 14-year-old girl with ntSCI, with the absence of MRI abnormality, exhibiting left-dominant paralysis after running shuttles. To the best of our knowledge, no systematic studies have quantified the sensitivity or detection rate of SEPs in SCIWORA, particularly in pediatric populations. Acknowledging this gap would better position the current findings as preliminary and hypothesis-generating. We conclude that SEPs could be an adjunctive method for detecting occult spinal lesions in children, especially in cases where spinal MRI fails to demonstrate the lesion responsible for ntSCI.
